# Green Tea Catechin, Epigallocatechin Gallate, Suppresses Signaling by the dsRNA Innate Immune Receptor RIG-I

**DOI:** 10.1371/journal.pone.0012878

**Published:** 2010-09-22

**Authors:** C. T. Ranjith-Kumar, Yvonne Lai, Robert T. Sarisky, C. Cheng Kao

**Affiliations:** 1 Department of Molecular and Cellular Biochemistry, Indiana University, Bloomington, Indiana, United States of America; 2 Pharmaceutical Research & Development, Johnson & Johnson, Raritan, New Jersey, United States of America; Cleveland Clinic, United States of America

## Abstract

**Background:**

The Innate immune system constitutes the first line of defense against pathogen infections. The Retinoic acid-inducible gene I (RIG-I) receptor recognizes triphosphorylated ssRNAs and dsRNA to initiate downstream signaling of interferon response. However, unregulated activity of these receptors could lead to autoimmune diseases. We seek to identify small molecules that can specifically regulate RIG-I signaling.

**Methodology/Principal Findings:**

Epigallocatechin gallate (EGCG), a polyphenolic catechin present in green tea, was identified in a small molecule screen. It was found to bind RIG-I and inhibits its signaling at low micromolar concentrations in HEK293T cells. Furthermore, EGCG dose-dependently inhibited the ATPase activity of recombinant RIG-I but did not compete with RIG-I interaction with RNA or with ATP. EGCG did not inhibit signaling by Toll-like receptors 3, 4, 9 or constitutive signaling by the adapter protein IPS-1. Structure activity relationship analysis showed that EGCG, its epimer GCG and a digallate-containing compound, theaflavin 3,3′ digallate (TFDG) were potent RIG-I inhibitors. EGCG also inhibited IL6 secretion and IFN- β mRNA synthesis in BEAS-2B cells, which harbors intact endogenous RIG-I signaling pathway.

**Conclusions/Significance:**

EGCG and its derivatives could have potential therapeutic use as a modulator of RIG-I mediated immune responses.

## Introduction

Multiple, at least partially overlapping, pathways are used to detect viral infections [1]. During RNA virus infection, double-stranded RNAs (dsRNA) and uncapped transcripts generated during replication can serve as pathogen-associated molecular patterns recognized by innate immune receptors [1], [2]. Agonist binding by these receptors results in changes in signal transduction that can lead to establishment of antiviral responses as well as mediate adaptive immune responses. Improper regulation of signaling by these receptors could result in inflammation-associated pathologies. It is therefore important to be able to modulate the signal transduction by the innate immune receptors.

The Retinoic acid inducible gene I (RIG-I) encodes a receptor that recognizes cytoplasmic RNAs. The RIG-I protein contains two N-terminal caspase recruitment domains (CARD), a central DExD/H RNA helicase domain, and a C-terminal regulatory domain of 190 amino acids that interacts with the helicase domain and the CARD domain to suppress signaling [3]. Upon agonist binding by the regulatory and possibly the helicase domains, RIG-I undergoes a conformational change to expose the CARD domain and may also cause multimerization of RIG-I [3]. The exposed CARDs can then interact with and activate its adaptor protein IPS-1 (also known as MAVS, VISA and Cardif), a mitochondrial membrane protein, to activate signal transduction through the transcription factors IRF3 and NF-κB resulting in increases in cytokine production [4], [5].

Regulation of RIG-I signaling has been an area of intense focus in part because it can modulate the outcome of virus infection and also inflammation-associated diseases. Several types of ligands are recognized by RIG-I. Single- and double-stranded RNAs with a 5′ terminal triphosphate are specifically recognized by C-terminal regulatory domain [6]–[10]. This recognition could serve to discriminate between self (capped mRNAs) and nonself RNAs (that may not be capped). Highly structured portions within the hepatitis C virus (HCV) genomic RNA and the polyuridine motif of the 3′ untranslated region are also ligands for RIG-I [3], [11], [12]. RIG-I could also recognize blunt-ended dsRNA that lack a 5′ triphosphate due to the stacking of a phenylalanine in the C-terminal regulatory domain with the terminal base pair of the RNA [13], [14]; unpublished data]. Single-stranded DNAs containing phosphorothioates are also potent antagonists of RIG-I activation of signal transduction [13].

We seek to identify chemical modulators of RIG-I for use as tools to elucidate RIG-I's mechanism of action. A screen of a small molecule chemical library revealed that the Epigallocatechin-3-gallate (EGCG) could bind to RIG-I and modulate its signaling. EGCG is one of the four major polyphenolic catechins that are found in green tea. These catechins have been linked to important health benefits including reduced risk for atherosclerosis, cancer, oxidative damage from free radicals and cardiovascular diseases [15]–[17]. EGCG inhibited RIG-I signaling in a cell-based reporter assay and RNA-dependent ATPase activity of recombinant RIG-I protein in biochemical assays. In lung epithelial BEAS-2B cells, EGCG inhibited RIG-I-specific IL-6 production. The inhibitory effect of EGCG was also observed with MDA-5, but not observed with several Toll-like receptors. To our knowledge EGCG is the first natural small molecule to regulate RIG-I dependent signal transduction.

## Results

### EGCG inhibits RIG-I signaling

We used a cell-based RIG-I dependent luciferase reporter assay to identify modulators of RIG-I signaling. The RNA agonist used to induce RIG-I signaling was a single-stranded RNA with a 5′ triphosphate named shR9. A screen of ∼2000 compounds, from the NIH Clinical Collection (NCC), and commercial chemical libraries, tested at 10 µM yielded 75 potential activators or inhibitors of shR9-dependent RIG-I signal transduction (Figure S1A). To determine whether these compounds acted directly on RIG-I, or affected the signaling cascade downstream of RIG-I, we measured their effects on shR9 dependent RIG-I ATPase activity in a biochemical assay. Recombinant RIG-I exhibits ATPase activity only in the presence of agonists [13]. None of the putative activators of RIG-I signaling increased RIG-I ATPase activity in the absence or presence of RNA agonist. However, one inhibitor, EGCG, reduced shR9-dependent RIG-I ATPase activity (Figure S1B) and was chosen for further characterization.

**Figure 1:**
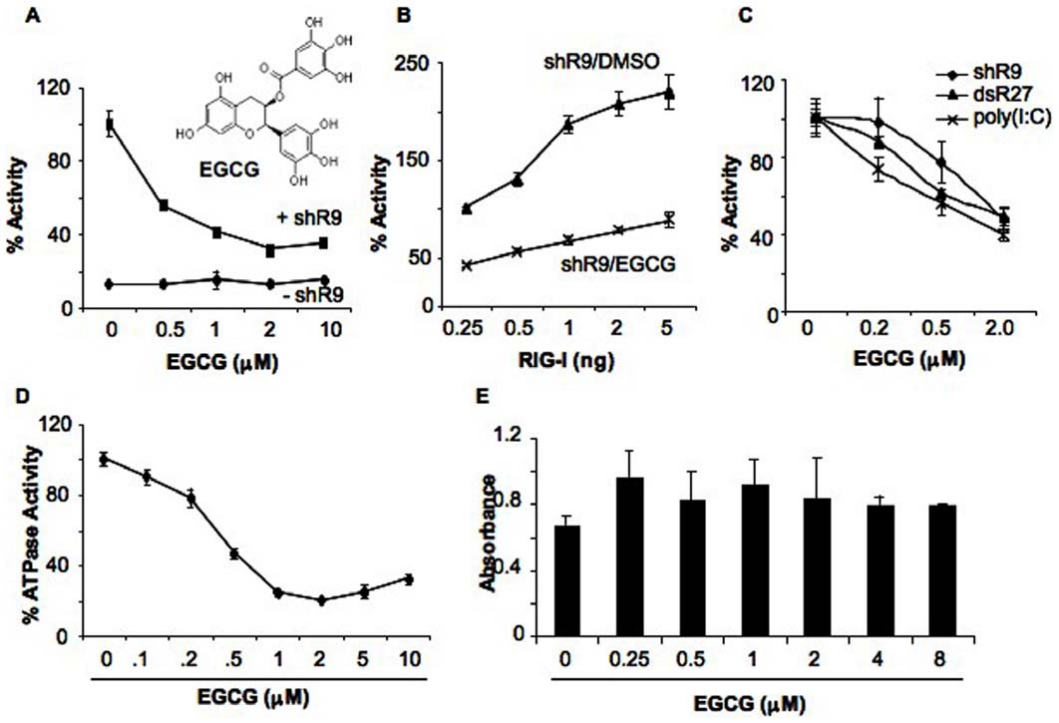
EGCG inhibits RIG-I signaling. (A) Effect of EGCG titration on triphosphorylated ssRNA, shR9-dependent RIG-I signaling. EGCG was added to HEK293T cells expressing full length RIG-I, IFN-β luciferase and *Renilla* luciferase reporters as described in the methods. The presence (+ shR9) and absence (- shR9) of shRNA shows the background and induced level of reporter expression in the presence of RIG-I. The activation of signaling obtained upon transfection of shR9 in the absence of EGCG was set as 100%. The chemical structure of EGCG is shown in the inset. The data are shown as a mean +/− standard deviation. (B) Effect of RIG-I plasmid concentration on EGCG inhibition. HEK293T cells were transfected with increasing amounts of pUNORIG-I plasmid that expresses full length RIG-I without changing the amount of reporter plasmids. The total amount of plasmids was kept constant by addition of pUNO vector plasmid. EGCG was added at 2 µM and DMSO was used as control. The data are represented as a mean +/− standard deviation. (C) Activation of RIG-I signaling with single and double stranded RNA agonists was inhibited by EGCG in the reporter assay in HEK293T cells. The data are shown as a mean +/− standard deviation. (D) EGCG inhibits ATPase activity of recombinant full length RIG-I. Different amounts of EGCG were added along with shR9 agonist to the ATPase reaction. ATPase activity obtained with DMSO was considered as 100%. (E) WST-1 assay to determine the toxicity of EGCG in HEK293T cells. The data are shown as a mean +/− standard deviation.

EGCG inhibited shR9-dependent RIG-I signaling in a concentration-dependent manner, with an IC_50_ between 1 to 2 µM in the cell-based reporter assay (Figure 1A). EGCG reduced RIG-I signaling at all concentrations of the transfected RIG-I plasmid tested (Figure 1B). Similar IC_50_ values were found with blunt ended dsRNA ligand of 27 bp (dsR27) and the heterogeneous poly(I∶C) (Figure 1C). In addition, similar results were obtained when the NF-κB-Luc and ISRE-Luc reporters were used instead of the IFN-β-luc reporter (Table 1).

**Table 1:** Specificity of EGCG inhibition.

	% Activity							
	RIG-I			TLR3		TLR4	TLR9	IPS-1
	IFN-β	NF-κB	ISRE	ISRE	NF-κB	NF-κB	NF-κB	NF-κB
EGCG (2 µM)	41 (1)	42 (2)	40 (1)	87 (7)	90 (5)	110 (8)	91 (12)	97 (8)
Clemizole (2 µM)	119 (5)	147 (17)	121 (26)	128 (17)	99 (2)	97 (11)	98 (5)	111 (6)

The % activity is the normalized signaling activity by the innate immune receptor or the adaptor IPS-1 observed in the presence of 2 µM of EGCG and Clemizole in the reporter assay using HEK293T cells. The numbers correspond to the mean of the experiment and the standard deviation is in parenthesis. Analysis was performed using firefly luciferase reporter driven by IFN-β, NF-κB or ISRE promoters as mentioned above the % activity.

EGCG inhibited ATPase activity induced by shR9 in vitro in a concentration-dependent manner, with an IC_50_ less than 0.5 µM (Figure 1D). Increasing the concentration of recombinant RIG-I protein in the presence of constant amount of EGCG and shR9 reduced the inhibitory effect of EGCG, likely by titrating away EGCG (Figure S1C). These results suggest that EGCG acts by binding to RIG-I or the RIG-I-agonist complex.

To validate EGCG as a RIG-I inhibitor that warrants further characterizations, its cytotoxicity was tested using the WST-1 assay that assesses for mitochondrial succinate-tetrazolium reductase in viable cells. EGCG had no significant affect on cell viability at concentration up to 10 µM, more than 10-fold of IC_50_ (Figure 1D and data not shown). The *Renilla* luciferase activity in the cell based reporter assays was also unaffected by up to 10 µM of EGCG (data not shown). EGCG thus has minimal cytotoxicity at the concentrations capable of inhibiting signal transduction by RIG-I.

### Specificity of EGCG on RIG-I

To test whether EGCG has specificity for RIG-I, we used luciferase reporter assays driven by NF-κB promoter elements that are responsive to TLR3, TLR4, TLR9 as well as RIG-I. At 2 µM, EGCG did not affect signaling by TLR3, TLR4 or TLR9, demonstrating a selective effect for RIG-I among the receptors tested (Table 1).

To confirm the specificity of EGCG, we expressed the RIG-I adaptor, IPS-1, from a strong promoter, which will activate ligand-independent signal transduction [18]. EGCG was unable to inhibit ligand-independent IPS-1-activated reporters, demonstrating that EGCG did not affect the signaling pathway downstream of IPS-1 activation (Table 1). Finally, another anti-inflammatory compound, Clemizole, did not significantly affect signaling by any of the receptors tested, including RIG-I. EGCG did inhibit MDA5 with an IC_50_ between 1 to 2 µM, similar to the IC_50_ of RIG-I (Figure S2). These results demonstrate that EGCG can specifically affect RIG-I-like receptors (Table 1).

### Effects of EGCG on ATP binding by RIG-I

**Figure 2:**
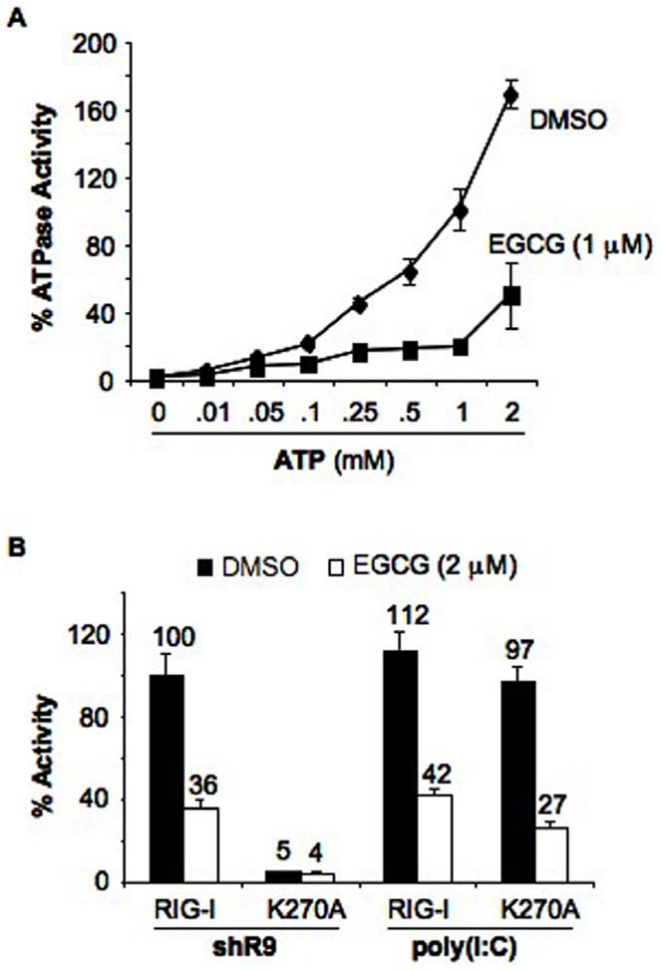
EGCG does not compete with ATP binding of RIG-I. (A) EGCG does not compete with ATP binding of RIG-I. Effect of ATP concentration on EGCG inhibition of recombinant full length RIG-I ATPase activity. Different amounts of ATP were added to the shR9 dependent ATPase activity in the presence of EGCG (1 µM) or DMSO. The data are shown as a mean +/− standard deviation. (B) Effect of EGCG on ATPase activity deficient mutant K270A in cell-based reporter assay. Signaling by WT and K270A mutant was analyzed using triphosphorylated RNA shR9 and dsRNA mimic, poly(I∶C) in the presence and absence of EGCG (2 µM). K270A was incapable of signaling with shR9 but could induce signaling with poly(I∶C). Signaling of WT RIG-I with shR9 was set as 100%. Assay was performed using IFN-β luciferase as reporter plasmid and the data are shown as a mean +/− standard deviation.

Shim et al. [19] reported that EGCG binds to ZAP-70 kinase at the catalytic site and blocks the ATP-binding pocket, prompting us to examine whether EGCG could have a similar effect on RIG-I. Increasing ATP concentration up to 2 mM resulted in a corresponding increase in RIG-I ATPase activity (Figure 2A). However, 1 µM of EGCG inhibited the ATPase activity of RIG-I to 30% in the presence of 2 mM ATP, suggesting that EGCG does not interfere with ATP binding site or acts on portions of RIG-I in addition to the ATP binding site. To confirm this, we tested RIG-I with a Walker A box mutation (K270A) that affected signaling by RIG-I in response to triphosphorylated ssRNA, but not to poly(I∶C) [13]. EGCG inhibited poly(I∶C)-dependent signaling of RIG-I K270A mutant to the same level as the wild type (Figure 2B). This result further supports our claim that EGCG is not acting as a competitive inhibitor of ATP-binding by RIG-I.

### EGCG and RNA-binding by RIG-I

**Figure 3:**
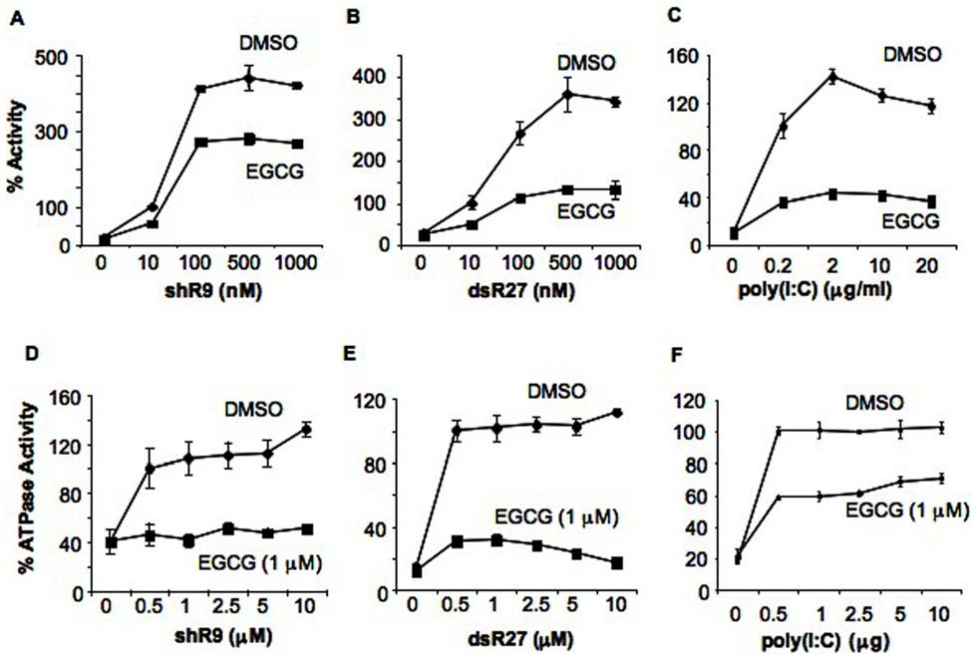
RIG-I RNA binding is not affected by EGCG. (A–C). Cell based reporter assay to analyze the effect of EGCG (2 µM) on full length RIG-I signaling with increasing concentrations of shR9 (A), blunt ended dsRNA, dsR27 (B) and poly(I∶C) (C). Signaling observed with 10 nM of shR9, dsR27 and 0.2 µg/ml of poly(I∶C) was considered as 100%. Assay was performed using IFN-β luciferase as reporter plasmid. All the data are shown as a mean +/− standard deviation. (D–F). Inhibition of full length RIG-I ATPase activity by EGCG was analyzed using different amounts of shR9 (D), dsR27 (E) and poly(I∶C) (F). ATPase activity obtained with 0.5 mM of shR9, dsR27, and 16 µg/ml poly(I∶C) was normalized to 100%. All data are shown as a mean +/− standard deviation.

We next examined whether EGCG could affect RNA binding by RIG-I. Should EGCG and the ligand compete for the same binding pocket, increasing the RNA concentration would affect the kinetics of EGCG inhibition. SsRNA and dsRNA were transfected into HEK293T cells expressing RIG-I at concentrations from 10 nM to 1 µM and approximately 100 nM of the triphosphorylated shR9 saturated RIG-I signaling (Figure 3A). EGCG at 2 µM reduced RIG-I signaling induced by 10 nM RNA. Notably, a ten-fold higher shR9 was unable to reverse the inhibitory effect of EGCG, indicating that EGCG is not a competitive inhibitor of the RIG-I recognition of triphosphorylated RNA (Figure 3A). Similar results were obtained with dsR27 and poly(I∶C) (Figure 3B & 3C).

Several mutations in the RIG-I regulatory domain that affected RNA binding have been characterized [6], [7], [14]. Mutants, H847A, K858A, and K861A all exhibited varying degrees of reduced RIG-I-dependent signal transduction (Figure S3). However, EGCG at 2 µM was able to further reduce reporter activation by all of three mutants (Figure S3).

The ATPase assay was used to examine whether RNA and EGCG have distinct binding requirements. Triphosphorylated ssRNA and dsRNA were added to the assay while EGCG and protein concentrations were kept constant (Figure 3D–F). Both shR9 and dsR27 at 10 µM and poly(I∶C) at 10 µg were unable to overcome the inhibition by 1 µM of EGCG. All the results from Figure 3 and Figure S3 suggest that if EGCG is competing for RNA binding to RIG-I, it has a higher affinity than the RNA.

### Effects of EGCG on RIG-I deletion mutants

**Figure 4:**
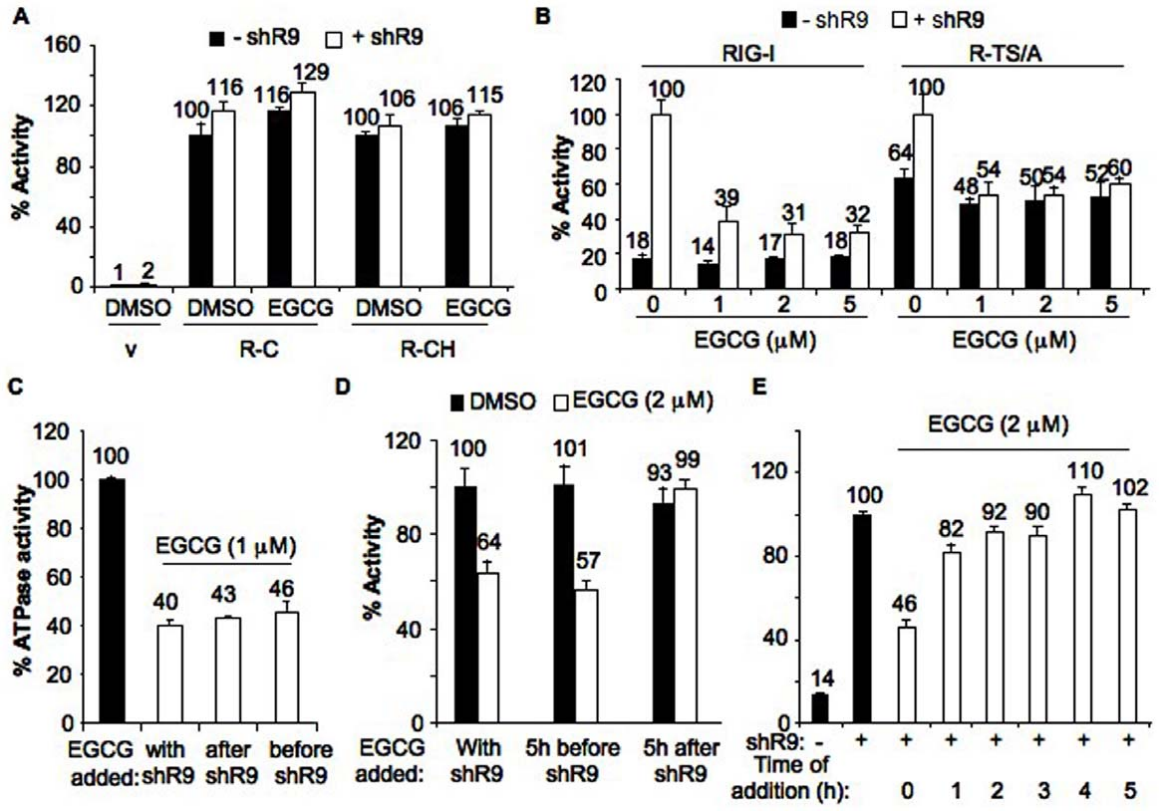
Mode of action of EGCG. (A) Constitutively active RIG-I mutants were not inhibited by EGCG. RIG-I deletion mutants expressing only the dual CARD domains, R-C and CARD-helicase domains, R-CH was analyzed using the cell based reporter assay in the presence of EGCG (2 µM) or DMSO. Black and white bars correspond to signaling activity observed in the absence and presence of transfected shR9. The data are shown as a mean +/− standard deviation. (B) EGCG inhibits only the agonist-dependent signaling of RIG-I. Cell based reporter assay was performed to determine the effect of EGCG on WT RIG-I and mutant R-TS/A with (white bars) and without (black bars) transfected shR9. Amount of EGCG used is shown below the x-axis. The data are shown as a mean +/− standard deviation. (C) ATPase activity and order of addition of EGCG. EGCG was incubated with recombinant full length RIG-I either together, 10′ before or after shR9. ATP was then added to initiate ATPase activity. The data are shown as a mean +/− standard deviation. (D and E) RIG-I cell based assay and order of EGCG addition. EGCG was added together, 5 h before or after transfection of shR9 into cells (D) or after every 1 h interval post transfection up to 5 h (E). EGCG failed to inhibit RIG-I signaling when added 1 h post transfection of shR9. All assays were performed using IFN-β luciferase as reporter plasmid and the data are shown as a mean +/− standard deviation.

To test potential interaction site of EGCG in RIG-I, we tested RIG-I deletion mutants that can activate signal transduction in the absence of exogenous ligands. The deletion mutant R-C harbors the two CARD domains (N terminal 228 a.a.) while the R-CH mutant contains the CARD and the helicase domains (N-terminal 734 aa). Both mutants exhibit ligand-independent signal transduction (Figure 4A). Not surprisingly, transfection of shR9 did not significantly induce signaling by R-C and R-CH. EGCG did not inhibit constitutive signaling of R-C and R-CH, suggesting that it does not interact with the CARD domain or the helicase domain (Figure 4A).

A RIG-I double mutant R-TS/A (T409A/S411A) also exhibited enhanced ligand-independent activity when compared to wild-type RIG-I [13] (Figure 4B). R-TS/A retained the ability to respond to agonists, whose presence resulted in an elevated reporter expression. EGCG inhibited shR9-dependent signaling of R-TS/A similar to the level of WT RIG-I but did not significantly affect ligand-independent constitutive signaling (Figure 4B). Taken together with the results from the analysis of effects with the ligand-binding pocket and the ATPase pocket, we concluded that EGCG could act on the ligand-bound complex of RIG-I.

To further confirm this hypothesis, we assessed the effects of the time of EGCG addition on shR9–dependent RIG-I ATPase activity. RIG-I was incubated with EGCG and RNA together, or with EGCG 10 minutes before or after RNA, prior to ATP addition (Figure 4C). Similar levels of inhibition were observed when EGCG was added with, before, or after, the RNA. These results show that EGCG could inhibit RIG-I after RNA binding, although it doesn't inform us as to whether RNA remains bound after EGCG binding. In the cell-based reporter assay, EGCG added together with transfected shR9, or 5 h prior to RNA transfection, resulted in a similar degree of inhibition (Figure 4D). However, EGCG added 1 or 5 h after shR9 failed to inhibit RIG-I (Figure 4D and 4E). This result is expected in the cell-based assays, since signaling should be rapid after the agonist-bound RIG-I interacted with IPS-1 and EGCG added after signaling is initiated is unlikely to inhibit the process. Furthermore, EGCG does not inhibit IPS-1 or an event downstream of IPS-1 (Table 1). Taken together all these results suggest that EGCG can bind to an RNA-bound conformer of RIG-I and prevent signaling.

### Structure Activity Relationship

**Figure 5:**
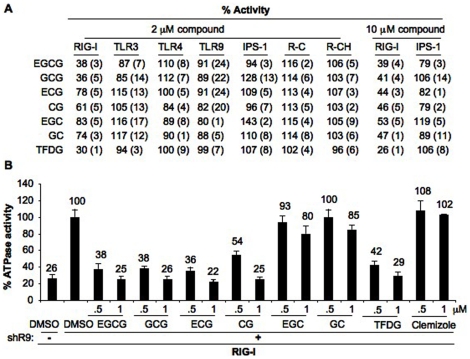
Structure activity relationship of EGCG. (A) Cell based reporter assay was used to test the effect of EGCG analogs (2 µM) on RIG-I, TLR3, TLR4, TLR9, IPS-1, R-C, and R-CH. The effect of 10 µM of these compounds was tested on RIG-I and IPS-1. The numbers correspond to mean percent activity in comparison to DMSO treated sample (taken as 100%). Standard deviation is given in parenthesis. TLR3, 4 & 9 assays used NF-κB-luciferase as reporter plasmid and all others were performed with IFN-β luciferase as reporter plasmid. (B) Analysis of EGCG analogs on shR9 dependent RIG-I ATPase activity. Each compound was tested at 0.5 and 1 µM. Clemizole was used as a control compound. The data are shown as a mean +/− standard deviation.

To better understand how EGCG structure relates to its ability to inhibit RIG-I, we tested several commercially available analogs, including epicatechin gallate (ECG), epigallocatechin (EGC) and the epimers gallocatechin gallate (GCG), catechin gallate (CG), gallocatechin (GC), and theaflavin 3,3′ digallate (TFDG) (Figure S4). At 2 µM, EGCG, its epimer GCG, and the digallate TFDG reduced RIG-I signaling by more than 60% (Figure 5A). TFDG inhibited RIG-I signaling with IC_50_ value of approximately 1 µM (data not shown). All the other analogs were less effective, reducing RIG-I signaling by 20–40%. The analogs did not significantly affect signaling by TLR3, TLR4, TLR9, or the constitutive activities of IPS-1, R-C and R-CH (Figure 5A). However, at 10 µM all the EGCG analogs showed similar levels of inhibition of RIG-I signaling but did not affect IPS-1 constitutive activity, suggesting that the less effective inhibitors either had poorer affinity for RIG-I or less optimal pharmacokinetic properties (Figure 5A).

In the biochemical ATPase assay, GCG, ECG, CG and TFDG inhibited the shR9-induced RIG-I ATPase activity to similar degree as EGCG (Figure 5B). EGC and its epimer GC as well as the control compound Clemizole did not show significant inhibition at the two concentrations tested (Figure 5B). These results show that analogs can bind recombinant RIG-I with similar properties and that pharmacokinetic properties is the more likely explanation for the differences in the results for the cell-based assays.

### Binding of EGCG to RIG-I

**Figure 6:**
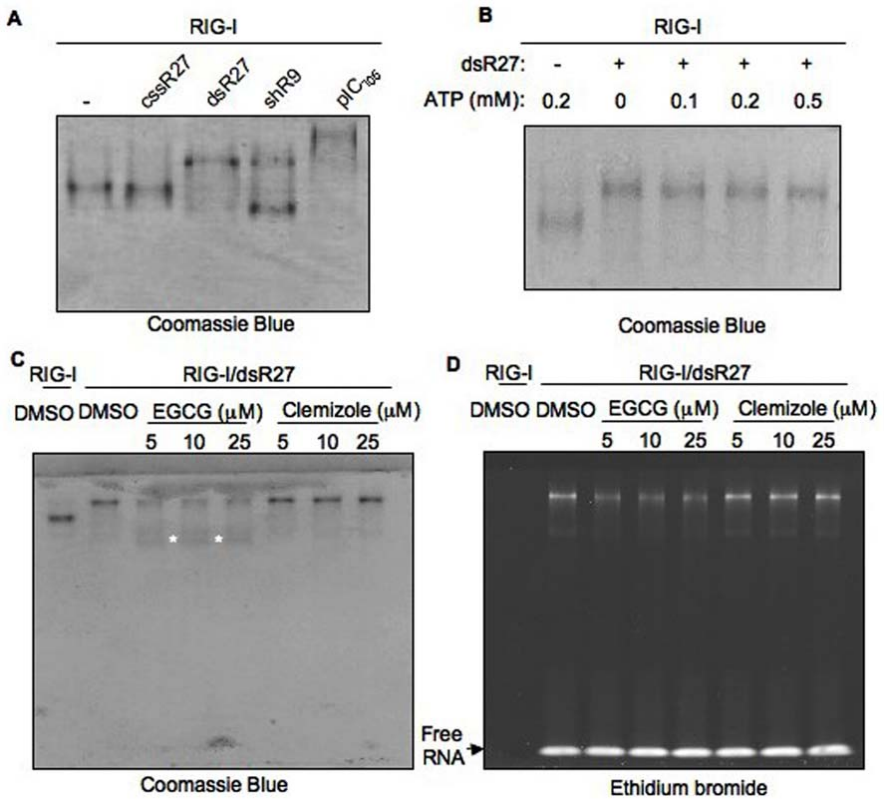
Native PAGE analysis of RIG-I-EGCG interaction. (A) Recombinant RIG-I was incubated with different RNAs shown above the gel and stained with Coomassie brilliant blue to detect RIG-I protein as indicated below the gel image. cssR27 is an unphosphorylated 27-mer ssRNA, dsR27 is 27-mer blunt ended dsRNA, shR9 triphosphorylated 60-mer short hairpin RNA and pIC_105_ is poly(I∶C) of average length of 105 bp. A shift in RIG-I mobility was observed only with RNAs that induced RIG-I signaling and ATPase activity. (B) Mobility shift does not require ATP. RIG-I was incubated with or without dsR27 in the absence or presence of different amounts of ATP (given on top of the gel). The presence of dsR27 is denoted with + sign above the gel. The gel is stained with Coomassie brilliant blue. (C) EGCG induces different mobility of RIG-I. Increasing concentrations of EGCG and Clemizole were added to RIG-I-dsR27 complex. The presence dsR27 and identity of the compounds are given on the top of the gel. The gel is stained with Coomassie blue and the fainter material with altered electrophoretic mobility is identified with white asterisks. (D) Same as (C) except the gel is stained for RNA using ethidium bromide.

RIG-I can bind to select RNAs in a native gel electrophoresis [7]. To further explore the potential RIG-I EGCG interaction we analyzed the RIG-I-agonist complexes using an electrophoretic mobility shift assay, followed by staining for the protein (Figure 6A). RIG-I incubated with dsR27, but not with unphosphorylated ssRNA, cssR27, exhibited a change in electrophoretic mobility from the apoprotein. This suggests that the RIG-I interacts with dsR27 but not with cssR27. A change in electrophoretic property of RIG-I was also observed with shR9 and poly(I∶C) of ∼105 bps (Figure 6A). Interestingly, shR9 bound RIG-I migrated as two distinct bands. This alternate mobility could be due to the different conformations of the complex formed between RIG-I and triphosphorylated RNA, shR9. These results confirm that RIG-I interacts only with RNAs that are agonists for signal transduction. Notably, ATP was not required for this complex formation (Figure 6B).

The mobility shift assay was used to examine the effect of EGCG on the RIG-I-dsR27 complex (Figure 6C). The addition of EGCG, but not Clemizole, resulted in altered mobility of the RIG-I-dsR27 complex. Along with the protein band with decreased mobility, protein band with increased mobility (indicated by asterisks in Fig. 6B) was also observed in the presence of EGCG but not Clemizole. To determine whether dsR27 is associated with RIG-I, the gel was stained with ethidium bromide (Figure 6D). A corresponding decrease in protein and RNA was observed with increasing amounts of EGCG but not with Clemizole. The RIG-I-EGCG complex migrated with increased mobility but did not get stained with ethidium bromide. No detectable RNase or protease activity was observed when EGCG was added to RIG-I-RNA complex (data not shown). These results suggests that the binding of EGCG to RIG-I-RNA complex resulted in the release of RNA agonist form the complex leading to a RIG-I-EGCG complex that is inactive for signaling. A similar change was also observed with TFDG (data not shown).

### EGCG and IL6 production in BEAS-2B cells

**Figure 7:**
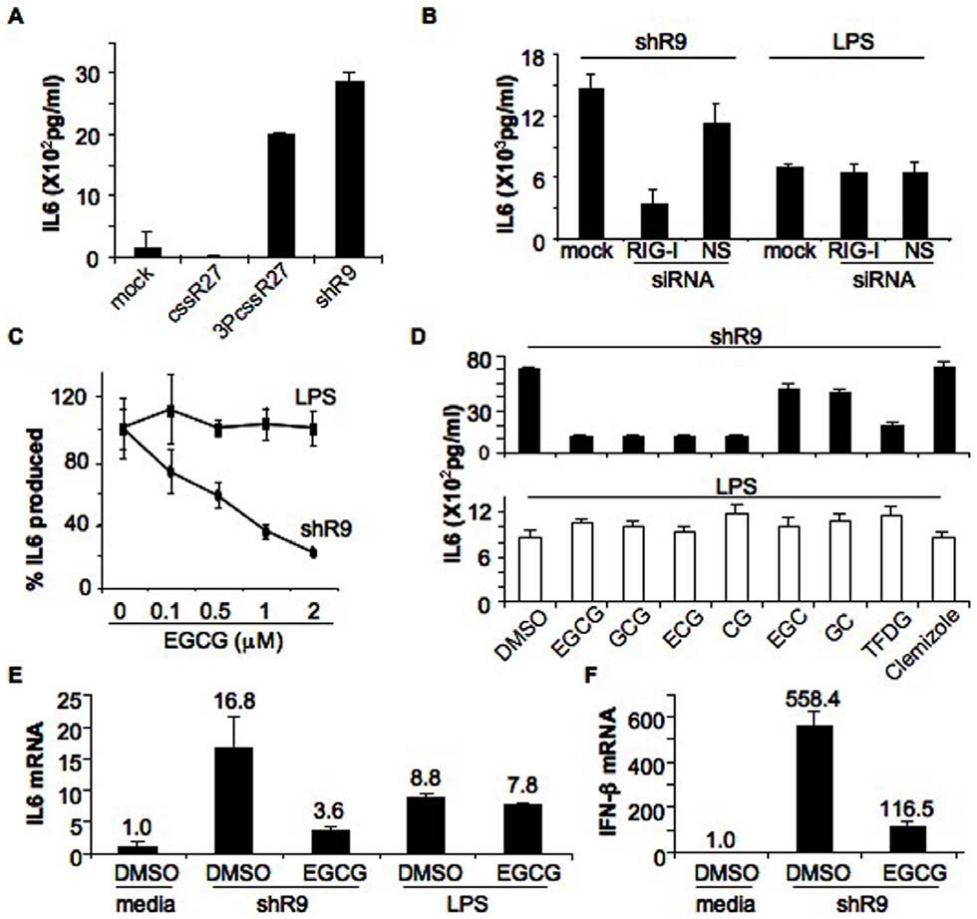
Effect of EGCG in BEAS-2B cells. (A) IL6 secretion by BEAS-2B cells was analyzed after transfection with different RNAs. Mock corresponds to no RNA transfected, cssR27 is an unphosphorylated 27-mer ssRNA, 3PcssR27 is same as cssR27 but with 5′ triphosphates and shR9 triphosphorylated 60-nt short hairpin RNA. The data are shown as a mean +/− standard deviation. (B) IL6 production induced by transfection of shR9 is via recognition by RIG-I. BEAS-2B cells were mock transfected or transfected with siRNA to RIG-I or control siRNA (NS). These cells were either transfected with shR9 or treated with LPS to induce secretion of IL6. The data are shown as a mean +/− standard deviation. (C) EGCG inhibits shR9 dependent IL6 production but LPS dependent IL6 production is unaffected. IL6 secretion in the absence of EGCG is normalized to 100%. The concentration of EGCG used is shown below the x-axis. The data are shown as a mean +/− standard deviation. (D) EGCG analogs and IL6 production. Effect of EGCG analogs on IL6 production by transfected shR9 (top) and added LPS (bottom) was analyzed. The analogs used are given at the bottom of the graph. Clemizole was used as the control. The data are shown as a mean +/− standard deviation. (E) Real time PCR analysis to determine the mRNA levels of IL6. Fold activation of IL6 mRNA was determined using quantitative RT-PCR. Data were normalized to the gylceraldehyde-3-phosphate dehydrogenase levels in each sample and reported as fold induction of non-stimulated control cells. The data are shown as a mean +/− standard deviation. (F) EGCG inhibited IFN-β mRNA accumulation. IFN-β was analyzed in the presence and absence of EGCG in shR9 or mock transfected BEAS-2B cells and represented as given in (E). The data are shown as a mean +/− standard deviation.

Thus far, all of the cell-based effects of EGCG on RIG-I were analyzed with RIG-I expressed by transient transfection and overexpression of RIG-I in HEK293T cells. To determine whether endogenously expressed RIG-I is susceptible to inhibition by EGCG, we used a human bronchial epithelial cell line, BEAS-2B. IL-6 production by BEAS-2B cells increased in response to transfected triphosphorylated RNAs (shR9 and 3PcssR27), but not to RNAs lacking a 5′ triphosphate (cssR27) (Figure 7A). Furthermore, shR9-induced IL6 production was significantly decreased in the presence of siRNA against RIG-I but not with the control nonspecific siRNA (NS), demonstrating that signaling required RIG-I (Figure 7B). BEAS-2B cells can also induce IL-6 production upon activation with lipopolysaccharide (LPS) through TLR4 [20]. RIG-I siRNA did not affect LPS-induced IL6 production (Figure 7B). To ensure that the effects of EGCG are not as a result of cytotoxicity, we used a WST-1 assay to measure the effects of EGCG on the cell viability of BEAS-2B cells. EGCG, up to 8 µM did not show significant toxicity (Figure S5). These results demonstrate that IL-6 production by BEAS-2B cells is suitable for analysis of the effects of EGCG on endogenously produced RIG-I.

EGCG inhibited shR9-induced IL6 production in BEAS-2B cells in a concentration-dependent manner. EGCG also had only minimal effects on LPS-induced IL-6 production (Figure 7C). Some EGCG analogs also had inhibitory effects on shR9-induced IL-6 production, with GCG, ECG, CG and TFDG inhibiting IL6 production better than did EGC and GC (Figure 7D top). Clemizole, which did not affect RIG-I-dependent ATPase activity and reporter activity in the HEK293T cells, did not inhibit shR9-induced IL-6 production. No significant affect of LPS induced IL6 production was observed with any of the EGCG analogs (Figure 7D bottom).

To confirm that the effects of EGCG are at the level of transcriptional regulation in BEAS-2B cells, we analyzed the production of IL6 mRNA by real-time PCR (Figure 7E). EGCG at 1 µM inhibited shR9-dependent IL6 mRNA synthesis by 80% but did not significantly affect LPS-dependent IL6 mRNA synthesis. Furthermore, EGCG inhibited shR9-dependent IFN-β mRNA synthesis by 80% (Figure 7F). These results demonstrate that EGCG modulated a RIG-I –dependent change in signal transduction in a cell line with endogenous RIG-I pathway.

## Discussion

The innate immune response provides the first line of defense against invading pathogens. However, hyperactivity of these receptors could lead to autoimmune disorders. In our attempt to identify small molecule modulators of RIG-I we identified a green tea polyphenol, EGCG, which could bind RIG-I and inhibit its activation of signal transduction. EGCG did not interfere with RNA or ATP binding by RIG-I and can affect the complex of RIG-I and RNA (Figure 2, 3 and 6).

Interestingly, EGCG did not inhibit the constitutive signaling activity of RIG-I (Figure 1A). The R-TS/A mutant showed elevated ligand-independent signaling than WT RIG-I, but retained response to ligand, suggesting that the mutations have resulted in relaxed repression of CARD domain. Increasing amounts of EGCG inhibited only the agonist-dependent signaling but not the constitutive signaling by the TS/A mutant (Figure 4B). Furthermore, EGCG did not inhibit the constitutive activities of RIG-I deletion mutants R-C and R-CH (Figure 4A). These results suggest that EGCG targets a conformer of the RIG-I complexed with its agonist. Pre-treatment of cells with EGCG did not augment inhibition of signaling or ATPase activity, suggesting that EGCG did not prevent RNA ligand binding by RIG-I (Figure 2C–E). However, the RNA signal normally present in complex with RIG-I was decreased in the presence of EGCG, suggesting that EGCG may be able to evict some RNAs in the RIG-I complex (Fig. 7D). We note that EGCG does not inhibit fluorescence of ethidium bromide (Fig. 7D and data not shown). Finally, the cell-based results are consistent with the biochemical results showing that EGCG and some analogs can alter RNA-dependent ATPase activity of RIG-I as well as the electrophoretic mobility of the RIG-I-agonist complex (Figure 5 and 6).

In HEK293T cells over-experessing RIG-I and other related innate immunity receptors, EGCG inhibited signaling by RIG-I and MDA-5 but not by Toll like receptors 3, 4 and 9 (Table 1 and Figure S2). In BEAS-2B cells expressing endogenous RIG-I and TLR4, only the shR9-induced and not the LPS-induced IL-6 production was inhibited by EGCG and its analogs (Figure 7). Finally, constitutive signal transduction by over-expressed IPS-1 was not affected by EGCG, indicating that EGCG does not act downstream of the IPS-1 in this signaling pathway (Table 1). We note that these experiments involve over-expression of IPS-1 and hence is not intended to provide a physiological condition, only to examine whether EGCG can suppress signaling downstream of IPS-1.

### Effects of EGCG on other targets

EGCG has been shown to interact with several proteins including fibronectin, fibrinogen, laminin, laminin receptor, glucose-regulated protein 78, ZAP-70 kinase etc [19], [21]–[25]. EGCG was also reported to bind to T-cell receptor CD4 and hence could have some potential for HIV therapy [26]. Reportedly EGCG inhibited TLR2 and TLR4 pathways in dental pulp cells and gastric epithelial cells [27], [28]. Furthermore, EGCG has been reported to inhibit the activation of transcription factor, NF-κB (possibly through the suppression of IκB kinase) and IRF3 (via suppression of kinase activity of TBK1) [29]–[32]. Our data are in contrast with the latter reports, as we did not see any significant inhibition of TLR3, TLR4, TLR9 and IPS-1 signaling measured via NF-κB reporters in HEK293T cells over expressing these receptors (Table 1). Furthermore, IL6 production in BEAS-2B cells in response to LPS treatment was unaffected. The discrepancy could be due to the amount of EGCG used in these studies. The IC_50_ for both RIG-I dependent cell-based reporter assays and ATPase assays was less than 2 µM of EGCG while the other studies had used EGCG at more than 50 µM for significant inhibition. We also observed that some EGCG analogs had activities at 10 µM while EGCG and TFDG were effective at ∼1 µM (Figure 5A). Thus it is possible that additional proteins will be affected by higher EGCG concentrations.

### Interpretation of SAR results

Analysis of EGCG agonists showed that EGCG and its epimer GCG are the most potent inhibitors among the analogs tested (Figure 5). Interestingly, there seems to be a difference in requirement for the inhibition of ATPase activity and signaling. EGCG, ECG and its epimers inhibited ATPase activity significantly while EGC and GC did not (Figure 5). This suggests that the cathechin gallate group participates in inhibition of ATPase activity. To inhibit RIG-I signaling, the three adjacent hydroxyl groups in the pyrogallol and galloyl group of the molecule appear to be required. However, it is possible that the difference is due to variations in the reaction conditions of the in vitro ATPase assay and cell-based reporter assay.

### Mechanism of EGCG inhibition

**Figure 8:**
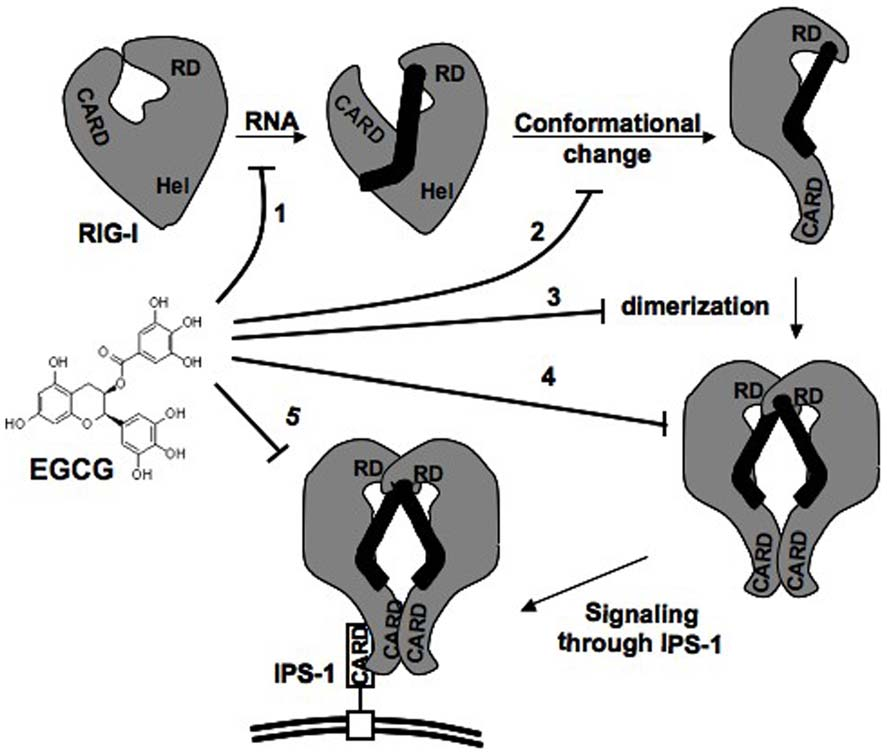
A model depicting RIG-I activation of signal transduction and the potential site of action of EGCG. Different domains of RIG-I depicted are, CARD (N-terminal dual CARD domains), Hel (helicase domain), RD (C-terminal Regulatory domain). Different numbers correspond to potential site of action of EGCG. IPS-1 is the adapter of RIG-I and is localized on the mitochondrial membrane (depicted as double line). More details can be found in the text.

RIG-I can be activated by phosphorylated ssRNAs, dsRNAs as well as non-phosphorylated dsRNAs [10], [13], [33]. Upon interaction with RNA, RIG-I undergoes a conformational change leading to dimerization of the RNA-bound protein (Figure 8). This complex then interacts with adaptor protein, IPS-1, which is present on the mitochondrion-associated membranes and peroxisomal membranes leading to downstream signaling [34], [35]. EGCG could interfere with this pathway at number of positions (Figure 8). First, EGCG could potentially bind to RIG-I and prevent its binding to the RNA agonist. This would sequester RIG-I in a state that cannot activate signal transduction. A second possibility is that EGCG could bind to RNA bound form of RIG-I and induce a different conformation resulting in the release of RNA. Third, EGCG could prevent the ligand-induced dimerization. A fourth possibility is that EGCG could bind to the RNA bound dimer of RIG-I and inhibit it from interacting with its adaptor protein, IPS-1. This complex may also be changed in the binding to the RNA and/or conformation upon binding of EGCG and the fifth possibility is that EGCG could interact with RIG-I-IPS-1 complex and prevent down stream signaling.

The time of addition experiment suggested that EGCG could inhibit RIG-I only after agonist binding and EGCG did not compete with RNA binding (Figure 4C–E). This suggests that EGCG did not prevent RNA binding thus ruling out scenario 1. Since, EGCG did not inhibit constitutive signaling by RIG-I mutants R-C, R-CH and R-TS/A (Figure 4A and 4B) it may not interfere with interaction of RIG-I with IPS-1 (scenario 5). Since EGCG does not compete with RNA binding (Figure 3), this could be interpreted as EGCG can bind to a conformer induced upon RIG-I-RNA interaction (scenario 2, 3 & 4). Native gel analysis (Figure 6C and 6D) suggested that EGCG binding to RIG-I-dsRNA complex resulted in the release of RNA from the complex (scenario 2). RIG-I bound to RNA is throught to occur as a dimer [3], [13], thus the inhibition is likely at scenario 4. While these studies provide several features for the interaction, additional studies will be required to elucidate of the mechanism(s) through which EGCG inhibits RIG-I signaling.

### Biomedical implications

The identification of EGCG inhibition of RIG-I activity provides proof of principle for small molecule modulators of this target. RIG-I is involved in sensing viral infections, including influenza virus and HCV [11], [36]. While RIG-I plays an active role in antiviral responses, these responses could also exacerbate pathologies. We note that infection of Epstein Barr virus (EBV) has been proposed to promote autoimmune diseases such as lupus [37]. EBV encoded small RNAs are transcribed by RNA polymerase III and are known to activate RIG-I [38]. In this light, EGCG could be a useful lead compound for additional engineering for both viral and autoimmune diseases. In addition, the identification of EGCG or its derivatives could be used as tool compounds to examine RIG-I signal transduction.

## Materials and Methods

### Constructs for expression in mammalian cells

The cDNA of RIG-I cloned in pUNO (pUNO-hRIG-I) was from Invivogen. Constructs expressing the RIG-I CARD (R-C; residues 1 to 229), and the CARD and helicase domains (R-CH; 1 to 734), were generated by amplifying the RIG-I cDNA sequence corresponding residues with flanking Age I and Nhe I restriction sites for cloning using these sites in the pUNO vector. All constructs were checked by DNA sequencing using the BigDye® Terminator v3.1 Cycle Sequencing Kits (Applied Biosystems, USA).

### Cell-based Reporter Assays

RIG-I assay was performed as per Ranjith-Kumar et al. [13] with IFN-β luciferase as the reporter plasmid (a kind gift from Dr. Rongtuan Lin, Lady Davis Institute for Medical Research, Montreal, QC, Canada.), unless mentioned otherwise. shR9 was transfected into the cells at 10 nM (final concentration). TLR3, TLR4 and TLR9 assays were performed as previously described [39] with pNiFty-Luc and the firefly reporter plasmid. Ligands for TLR3, 4 were 9 were, respectively, poly(I∶C) (500 ng/ml; Amersham Biosciences), lipopolysaccharide (1 µg/ml; Sigma) and ODN2006 (2 µM; Invivogen).

### Library screens

Compounds were analyzed using the cell-based assay described above. Compounds (dissolved in 10% DMSO) were added to the media prior to transfection of shR9 agonist. The final concentration of DMSO in each well was 0.2%. The ratio of firefly to *Renilla* luciferase values was calculated and the ratio obtained with agonist was considered as 100%. Compounds that increased or decreased signaling by 50% or more relative to DMSO controls are designated as activators and inhibitors, respectively.

### Expression and Purification of Recombinant RIG-I

RIG-I ORF was amplified and cloned into pET-28a vector at the Nde I and Xho I sites. The recombinant clone was confirmed by DNA sequencing and was transformed into Rosetta (DE3) PhyS cells. The cells were induced with 0.2 mM of IPTG for 12–16 h at 16°C and the RIG-I protein was first enriched using an Ni-NTA column which was pre-equilibrated with 20 mM Tris, pH 8.0, 300 mM NaCl, 10% glycerol, 10 mM imidazole, 5 mM beta-mercaptoethanol and the protein was eluted with equilibration buffer containing 500 mM imidazole. Fractions containing RIG-I were pooled, dialyzed against Buffer P containing 25 mM Tris, pH 8.0, 50 mM NaCl, 10% glycerol, and 10 mM beta-mercaptoethanol and purified by a Resource Q column (1 ml) that was pre-equilibrated with Buffer P. The protein was eluted in a buffer containing a gradient of 50 to 300 mM NaCl. Fractions containing purified protein were collected and dialyzed against buffer containing 25 mM Tris, pH 8.0, 175 mM NaCl, 10% glycerol and 10 mM beta-mercaptoethanol and stored at −80°C in aliquots.

### ATPase Assay

ATPase assay used the ADP-Glo Kinase Assay kit using the manufacturer's guidelines (Promega Inc). The reaction was performed in Buffer A: 20 mM Tris (pH 7.5), 5 mM MgCl_2_, 8 mM DTT, 30 mM NaCl and 4% glycerol that contained 1 mM ATP, 1 µM RNA (unless specified otherwise), and 100 nM of recombinant RIG-I protein unless stated otherwise. The samples were processed using ADP-Glo and Kinase detection reagent and luminescence was quantified using a Synergy 2 plate reader (Biotek Inc.). EGCG did not interfere with the luminescence detection.

### Electrophoretic mobility shift assays

Recombinant RIG-I (0.8 µM) was mixed with 4 µM RNA agonist and compounds (5–25 µM, DMSO was used for control), incubated at 37°C for 15 minutes, followed by addition of native PAGE loading buffer and separated on 10% PAGE at 4°C. The gel was then stained with ethidium bromide and/or Coomassie brilliant blue.

### WST-1 assay

To assess the effect of EGCG on cell viability of HEK293T and BEAS2B cells (ATCC), an in vitro colorimetric assay (WST-1 assay, Clontech, Mountain View, CA) was used. Cells were treated with 0–8 µM of EGCG (triplicates per dose). After 18 h, 10 µl of WST-1 reagent/100 µl medium was added to each well. After 1–6 h at 37°C, absorption was measured at 450 nm with 630 nm as reference using a microplate reader and corrected to blank values (wells without cells).

### Measurement of IL6 level in culture supernatant

Supernatants from BEAS2B cells were collected 24 h after treatments and stored at −20°C or assayed immediately for IL6 secretion using a human IL6 ELISA kit (R&D systems) as per manufacturer's protocol.

### RT-PCR

Total RNA was isolated from BEAS2B cells using the RNeasy Kit (QIAGEN, Valencia, CA) and digested with RNase-free DNase I (QIAGEN). RNA (0.5 µg) was then reversed transcribed to cDNA using Superscript III reverse transcriptase (Invitrogen, Carlsbad, CA) and random primers (New England Biolabs). Real-time PCR was performed using the Mastercycler *ep realplex* (Eppendorf). PCR amplications were performed in a total volume of 20 µl, containing 0.6 µl of cDNA and 200 nM of each primer, using SYBR Green I PCR reagents (Applied Biosystems). Data were normalized with gylceraldehyde-3-phosphate dehydrogenase levels in each sample and reported as fold induction of non-stimulated control cells. Primer sets: IL6 (sense, 5′CACAGACAGCCACTCACCTC-3′; anti-sense, 5′ AGCTCTGGCTTGTTCCTCAC-3′ IFN-β (sense, 5′-TGCTCTCCTGTTGTGCTTCTCC-3′; anti-sense, 5′-CATCTCATAGATGGTCAATGCGG-3′. GAPDH (sense, 5′-GAGTCAACGGATTTGGTCGT-3′; anti-sense, 5′-TGGGATTTCCATTGATGACA-

## Supporting Information

Figure S1 (A) Representative figure showing the cell based assay results of screening of certain compounds. The assay was performed using IFN-β luciferase as reporter. Asterisk depicts result obtained with EGCG. The data are shown as a mean +/− standard deviation. (B) Representative ATPase assay used for confirming cell based reporter assay result. Asterisk depicts result obtained with EGCG. 2006 corresponds to ODN2006, which was earlier shown to be a RIG-I inhibitor (Ranjith-Kumar et al., 2009). The data are shown as a mean +/− standard deviation. (C) Effect of RIG-I protein concentration on EGCG inhibition of shR9 dependent ATPase activity. The amount of RIG-I protein used is shown below the graph. Black and white bars correspond to DMSO and EGCG (1 µM) treatment respectively. ATPase activity observed with 100 nM RIG-I treated with DMSO was taken as 100%. The data are shown as a mean +/− standard deviation.
(0.05 MB PDF)

Figure S2 Effect of EGCG on MDA5 signaling. EGCG inhibited poly(I∶C) dependent MDA5 signaling in HEK293T cells with IFN-β luciferase as reporter. MDA5 signaling in the absence of EGCG was taken as 100%. The data are shown as a mean +/− standard deviation.
(0.03 MB PDF)

Figure S3 Cell based reporter assay using RIG-I RNA-binding mutants. RIG-I and mutant expressing HEK293T cells were mock transfected or transfected with shR9 in the presence of 2 µM of EGCG or DMSO. Assay was performed using IFN-β luciferase as reporter plasmid. Signaling activity observed with shR9 transfected WT RIG-I treated with DMSO was considered as 100%. The data are shown as a mean +/− standard deviation.
(0.03 MB PDF)

Figure S4 Chemical structures of EGCG analogs. Name of each analog is given under the structure.
(0.05 MB PDF)

Figure S5 Analysis of cytotoxicity of EGCG in BEAS-2B cells. WST-1 assay was used to determine the toxicity of EGCG in BEAS-2B cells. The amounts of EGCG added to the cells are given below the graph. The data are shown as a mean +/− standard deviation.
(0.03 MB PDF)
