# Supplementary figures and images for: Green Tea Catechin, Epigallocatechin Gallate, Suppresses Signaling by the dsRNA Innate Immune Receptor RIG-I
Source: PLoS One. 2010 Sep 22;5(9):e12878. doi: 10.1371/journal.pone.0012878 (PMC2943919; doi:10.1371/journal.pone.0012878)

Figure S1

**A**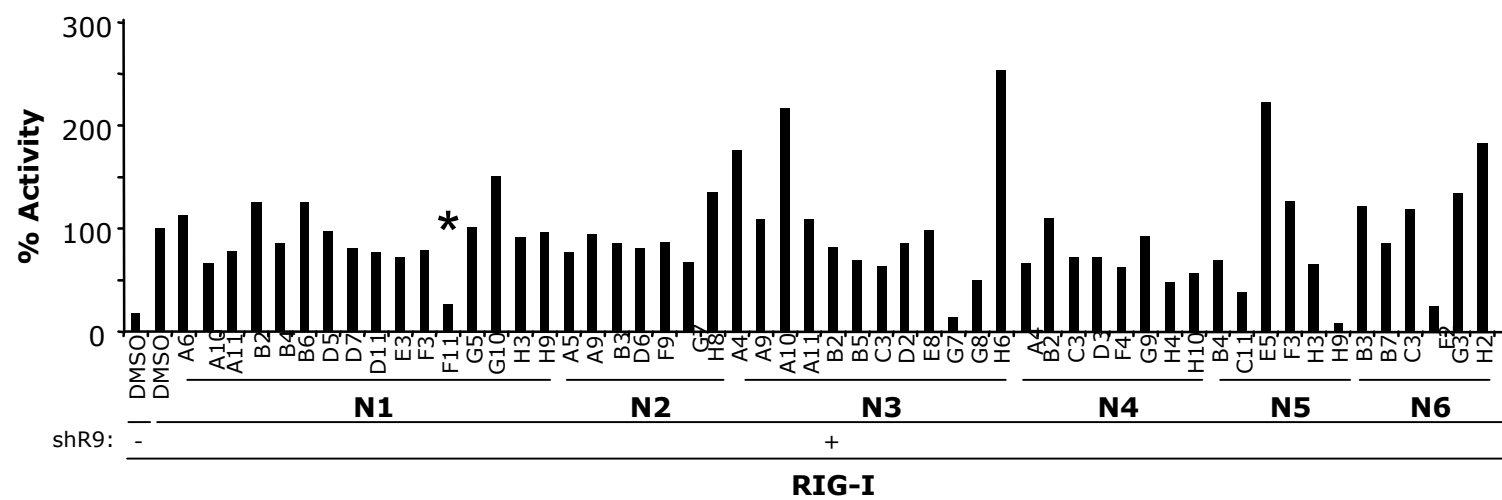**B**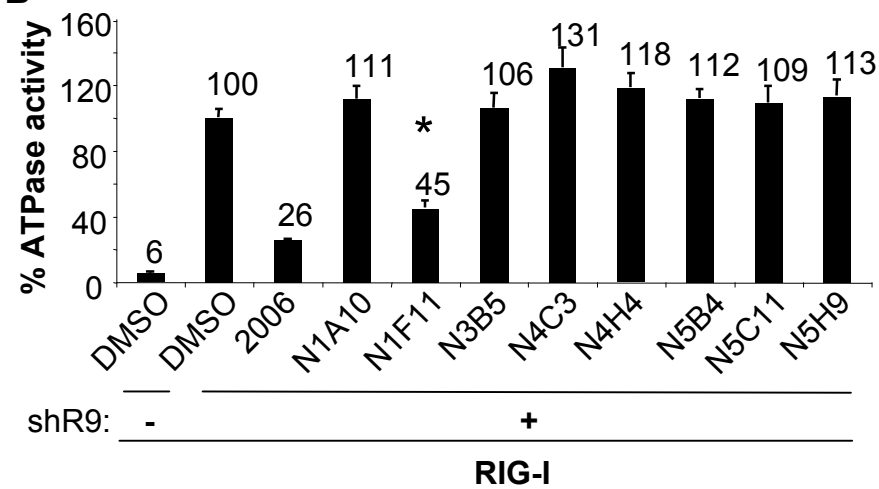**C**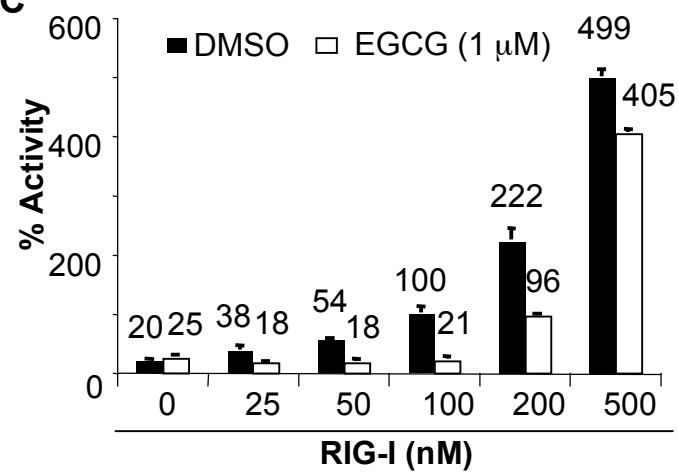

Supplement: Figure S1 — (A) Representative figure showing the cell based assay results of screening of certain compounds. The assay was performed using IFN-β luciferase as reporter. Asterisk depicts result obtained with EGCG. The data are shown as a mean +/− standard deviation. (B) Representative ATPase assay used for confirming cell based reporter assay result. Asterisk depicts result obtained with EGCG. 2006 corresponds to ODN2006, which was earlier shown to be a RIG-I inhibitor (Ranjith-Kumar et al., 2009). The data are shown as a mean +/− standard deviation. (C) Effect of RIG-I protein concentration on EGCG inhibition of shR9 dependent ATPase activity. The amount of RIG-I protein used is shown below the graph. Black and white bars correspond to DMSO and EGCG (1 µM) treatment respectively. ATPase activity observed with 100 nM RIG-I treated with DMSO was taken as 100%. The data are shown as a mean +/− standard deviation. (0.05 MB PDF) [file pone.0012878.s001.pdf]

Figure S2

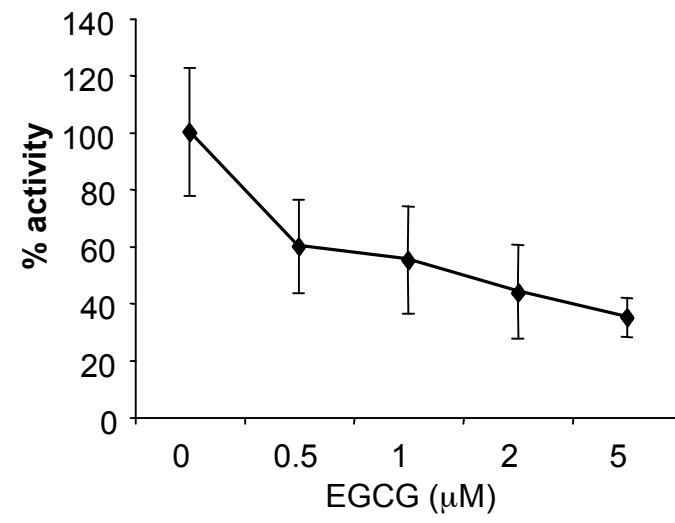

Supplement: Figure S2 — Effect of EGCG on MDA5 signaling. EGCG inhibited poly(I∶C) dependent MDA5 signaling in HEK293T cells with IFN-β luciferase as reporter. MDA5 signaling in the absence of EGCG was taken as 100%. The data are shown as a mean +/− standard deviation. (0.03 MB PDF) [file pone.0012878.s002.pdf]

Figure S3

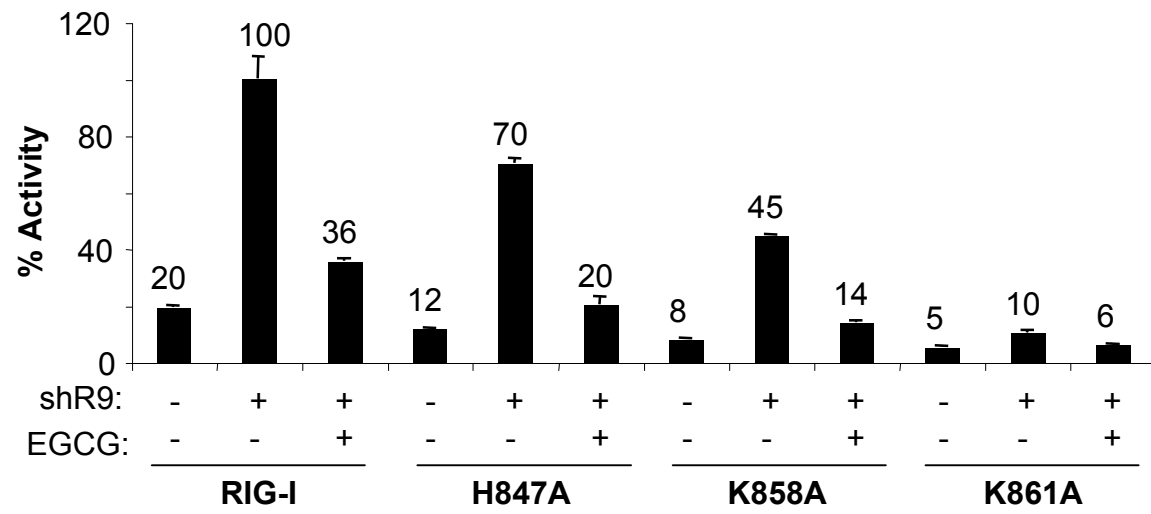

Supplement: Figure S3 — Cell based reporter assay using RIG-I RNA-binding mutants. RIG-I and mutant expressing HEK293T cells were mock transfected or transfected with shR9 in the presence of 2 µM of EGCG or DMSO. Assay was performed using IFN-β luciferase as reporter plasmid. Signaling activity observed with shR9 transfected WT RIG-I treated with DMSO was considered as 100%. The data are shown as a mean +/− standard deviation. (0.03 MB PDF) [file pone.0012878.s003.pdf]

Figure S5

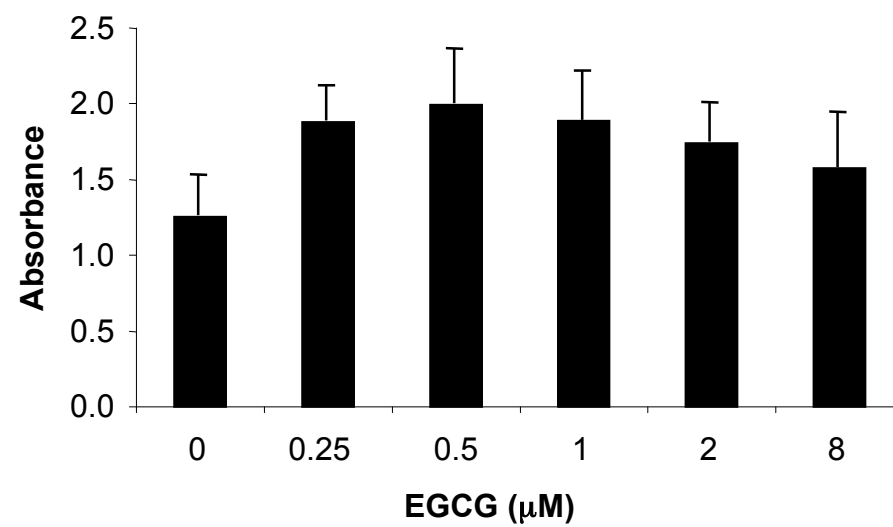

Supplement: Figure S5 — Analysis of cytotoxicity of EGCG in BEAS-2B cells. WST-1 assay was used to determine the toxicity of EGCG in BEAS-2B cells. The amounts of EGCG added to the cells are given below the graph. The data are shown as a mean +/− standard deviation. (0.03 MB PDF) [file pone.0012878.s005.pdf]
