# Supplementary material for: Green Tea Catechin, Epigallocatechin Gallate, Suppresses Signaling by the dsRNA Innate Immune Receptor RIG-I
Source: PLoS One. 2010 Sep 22;5(9):e12878. doi: 10.1371/journal.pone.0012878 (PMC2943919; doi:10.1371/journal.pone.0012878)
Supplement: Figure S4 — Chemical structures of EGCG analogs. Name of each analog is given under the structure. (0.05 MB PDF) [file pone.0012878.s004.pdf]

Figure S4

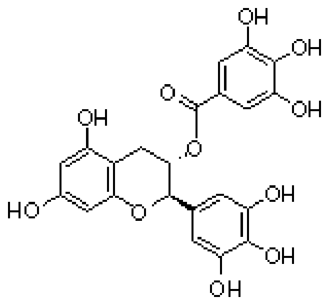

Gallocatechin gallate
(GCG)

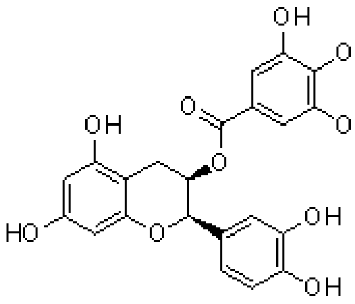

Epicatechin gallate
(ECG)

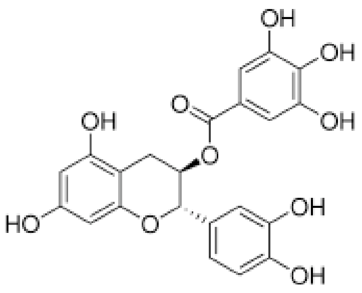

Catechin gallate
(CG)

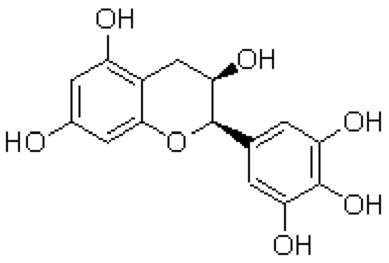

Epigallocatechin
(EGC)

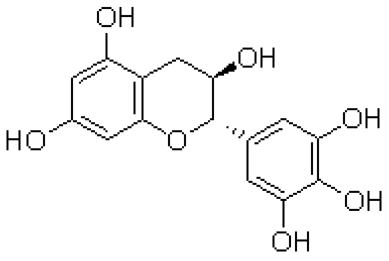

Gallocatechin
(GC)

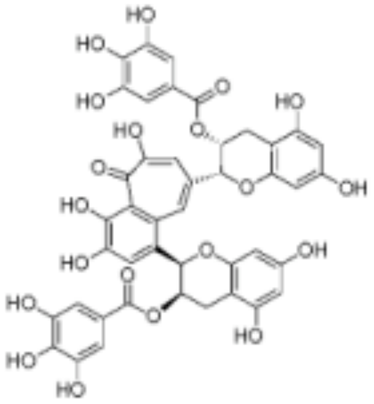

Theaflavin 3,3′-digallate
(TFDG)
